# *N*-(3-Chloro­phen­yl)-1,2-benzisothia­zol-3-amine 1,1-dioxide

**DOI:** 10.1107/S1600536810015163

**Published:** 2010-04-30

**Authors:** Tariq Saeed Shah, Waseeq Ahmad Siddiqui, M. Nawaz Tahir, Ghulam Hussain

**Affiliations:** a Department of Chemistry, University of Sargodha, Sargodha, Pakistan; b Department of Physics, University of Sargodha, Sargodha, Pakistan

## Abstract

In the title compound, C_13_H_9_ClN_2_O_2_S, the dihedral angle between the aromatic ring systems is 6.00 (12)° and an intra­molecular C—H⋯N inter­action generates an *S*(6) ring. In the crystal, mol­ecules inter­act by way of C—H⋯O and N—H⋯O bonds, generating *R*_2_^1^(7) and *R*_2_^2^(10) ring motifs, and aromatic π–π stacking inter­actions [centroid–centroid separations = 3.730 (3) and 3.733 (2) Å] help to consolidate the packing.

## Related literature

For other saccharin derivatives, see: Rafique *et al.* (2009 ▶); Siddiqui *et al.* (2010 ▶). For a related structure, see: Brigas *et al.* (2001 ▶). For graph-set theory, see: Bernstein *et al.* (1995 ▶).
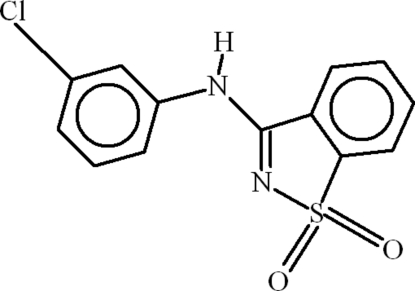


## Experimental

### 

#### Crystal data

C_13_H_9_ClN_2_O_2_S
*M* *_r_* = 292.73
Triclinic, 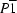

*a* = 7.2223 (10) Å
*b* = 7.9138 (12) Å
*c* = 11.2175 (17) Å
α = 96.178 (6)°
β = 98.840 (5)°
γ = 97.574 (5)°
*V* = 622.63 (16) Å^3^
*Z* = 2
Mo *K*α radiation
μ = 0.47 mm^−1^
*T* = 296 K
0.28 × 0.10 × 0.08 mm

#### Data collection

Bruker Kappa APEXII CCD diffractometer
Absorption correction: multi-scan (*SADABS*; Bruker, 2005 ▶) *T*_min_ = 0.947, *T*_max_ = 0.962
10481 measured reflections
2700 independent reflections
1244 reflections with *I* > 2σ(*I*)
*R*_int_ = 0.088

#### Refinement

*R*[*F* ^2^ > 2σ(*F* ^2^)] = 0.056
*wR*(*F* ^2^) = 0.115
*S* = 0.98
2700 reflections
172 parameters
H-atom parameters constrained
Δρ_max_ = 0.25 e Å^−3^
Δρ_min_ = −0.26 e Å^−3^

### 

Data collection: *APEX2* (Bruker, 2007 ▶); cell refinement: *SAINT* (Bruker, 2007 ▶); data reduction: *SAINT*; program(s) used to solve structure: *SHELXS97* (Sheldrick, 2008 ▶); program(s) used to refine structure: *SHELXL97* (Sheldrick, 2008 ▶); molecular graphics: *ORTEP-3 for Windows* (Farrugia, 1997 ▶) and *PLATON* (Spek, 2009 ▶); software used to prepare material for publication: *WinGX* (Farrugia, 1999 ▶) and *PLATON*.

## Supplementary Material

Crystal structure: contains datablocks global, I. DOI: 10.1107/S1600536810015163/hb5389sup1.cif

Structure factors: contains datablocks I. DOI: 10.1107/S1600536810015163/hb5389Isup2.hkl

Additional supplementary materials:  crystallographic information; 3D view; checkCIF report

## Figures and Tables

**Table 1:** Hydrogen-bond geometry (Å, °)

*D*—H⋯*A*	*D*—H	H⋯*A*	*D*⋯*A*	*D*—H⋯*A*
C13—H13⋯N2	0.93	2.28	2.901 (5)	124
N1—H1⋯O1^i^	0.86	2.24	3.084 (4)	165
C5—H5⋯O1^i^	0.93	2.51	3.396 (4)	159
C2—H2⋯O2^ii^	0.93	2.42	3.302 (4)	158

Symmetry codes: (i) 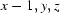; (ii) 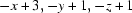.

**Fig. 1.**
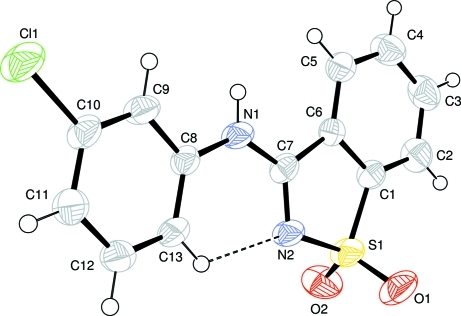
View of (I) with displacement ellipsoids drawn at the 50% probability level.

**Fig. 2.**
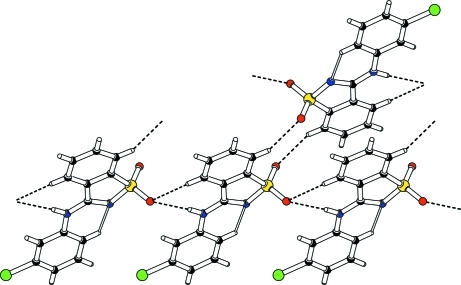
The partial packing of (I), which shows that molecules form polymeric sheets with various ring motifs.

## supplementary crystallographic information

### Comment

Due to the interest in obtaining new derivatives of saccharin (Rafique *et al.*, 2009: Siddiqui *et al.*, 2010), we wish to report the preparation and crystal structure of the title compound (I, Fig. 1).

The crystal structure of (II) *N*-(1,1-Dioxo-1,2-benzisothiazol-3-yl)-4- methoxyaniline (Brigas *et al.*, 2001) and (III) *N*-(1,1-Dioxo-1,2-benzisothiazol-3-yl)-3-methylaniline (Brigas *et al.*, 2001) have been published. The title compound differs from (II) and (III) due to attachement of chloro substition on the aniline. In (I), 1,2-benzisothiazol-3-amine A (C–C7/N2/S1) and 3-Chlorophenyl B (C8–C13/CL1) are planar with maximum r. m. s. deviations of 0.0080 Å and 0.0033 Å from the respective mean square planes. The dihedral angle between A/B is 6.00 (12)°. There exists an intramolecular H-bonding of C–H···N type forming S(6) ring motif (Bernstein *et al.*, 1995). The molecules are stabilized in the form of polymeric sheets due to intermolecular H-bondings (Table 1, Fig. 2) completing R_2_^1^(7) and R_2_^2^(10) ring motifs. There exist π–π interactions at a distance of 3.733 (2) Å and 3.730 (2) Å, between the centroids of the benzene rings Cg1 (C1—C6) and Cg2 (C8—C13) respectively, [Cg1···Cg1^i^: i = 2 - *x*,-*y*, 1 - *z*] and [Cg2···Cg2^ii^: ii = 1 - *x*,-*y*, - *z*].

### Experimental

A mixture of saccharin (1.0 g, 5.46 mmol) and *m*-chloroaniline (5 ml, in excess) was heated to reflux on an oil-bath (4 h), cooled to room temperature and kept overnight in a freezer. The solvent was evaporated under reduced pressure and the brownish yellow paste obtained was washed with benzene (4 × 25 ml) to obtain the bright light brown crystalline product (1.25, 78%, m. p. 578-579 K). Recrystallisation solvent: MeOH:AcOEt (1:1): the solution was subjected to slow evaporation at room temperature to obtain colourless needles of (I).

### Refinement

The H-atoms were positioned geometrically (C–H = 0.93, N–H = 0.86 Å) and refined as riding with *U*_iso_(H) = 1.2*U*_eq_(C, N).

### Crystal data

**Table:**

C_13_H_9_ClN_2_O_2_S	*Z* = 2
*M**_r_* = 292.73	*F*(000) = 300
Triclinic, *P*1	*D*_x_ = 1.561 Mg m^−^^3^
Hall symbol: -P 1	Mo *K*α radiation, λ = 0.71073 Å
*a* = 7.2223 (10) Å	Cell parameters from 1244 reflections
*b* = 7.9138 (12) Å	θ = 2.6–27.1°
*c* = 11.2175 (17) Å	µ = 0.47 mm^−^^1^
α = 96.178 (6)°	*T* = 296 K
β = 98.840 (5)°	Needle, colourless
γ = 97.574 (5)°	0.28 × 0.10 × 0.08 mm
*V* = 622.63 (16) Å^3^	

### Data collection

**Table:**

Bruker Kappa APEXII CCD diffractometer	2700 independent reflections
Radiation source: fine-focus sealed tube	1244 reflections with *I* > 2σ(*I*)
graphite	*R*_int_ = 0.088
Detector resolution: 7.60 pixels mm^-1^	θ_max_ = 27.1°, θ_min_ = 2.6°
ω scans	*h* = −8→9
Absorption correction: multi-scan (*SADABS*; Bruker, 2005)	*k* = −10→10
*T*_min_ = 0.947, *T*_max_ = 0.962	*l* = −14→14
10481 measured reflections	

### Refinement

**Table:**

Refinement on *F*^2^	Primary atom site location: structure-invariant direct methods
Least-squares matrix: full	Secondary atom site location: difference Fourier map
*R*[*F*^2^ > 2σ(*F*^2^)] = 0.056	Hydrogen site location: inferred from neighbouring sites
*wR*(*F*^2^) = 0.115	H-atom parameters constrained
*S* = 0.98	*w* = 1/[σ^2^(*F*_o_^2^) + (0.0363*P*)^2^] where *P* = (*F*_o_^2^ + 2*F*_c_^2^)/3
2700 reflections	(Δ/σ)_max_ < 0.001
172 parameters	Δρ_max_ = 0.25 e Å^−^^3^
0 restraints	Δρ_min_ = −0.26 e Å^−^^3^

### Special details

**Table:**

Geometry. All esds (except the esd in the dihedral angle between two l.s. planes) are estimated using the full covariance matrix. The cell esds are taken into account individually in the estimation of esds in distances, angles and torsion angles; correlations between esds in cell parameters are only used when they are defined by crystal symmetry. An approximate (isotropic) treatment of cell esds is used for estimating esds involving l.s. planes.
Refinement. Refinement of *F*^2^ against ALL reflections. The weighted *R*-factor *wR* and goodness of fit *S* are based on *F*^2^, conventional *R*-factors *R* are based on *F*, with *F* set to zero for negative *F*^2^. The threshold expression of *F*^2^ > σ(*F*^2^) is used only for calculating *R*-factors(gt) *etc*. and is not relevant to the choice of reflections for refinement. *R*-factors based on *F*^2^ are statistically about twice as large as those based on *F*, and *R*- factors based on ALL data will be even larger.

### Fractional atomic coordinates and isotropic or equivalent isotropic displacement parameters (Å^2^)

**Table:**

	*x*	*y*	*z*	*U*_iso_*/*U*_eq_	
C1	1.1663 (5)	0.2836 (4)	0.4969 (3)	0.0380 (9)	
C2	1.2431 (5)	0.3741 (5)	0.6082 (3)	0.0512 (11)	
H2	1.3719	0.4162	0.6278	0.061*	
C3	1.1225 (6)	0.4010 (5)	0.6905 (3)	0.0566 (12)	
H3	1.1705	0.4625	0.7668	0.068*	
C4	0.9329 (6)	0.3379 (5)	0.6609 (4)	0.0557 (11)	
H4	0.8542	0.3572	0.7177	0.067*	
C5	0.8563 (5)	0.2460 (5)	0.5481 (3)	0.0466 (10)	
H5	0.7275	0.2038	0.5289	0.056*	
C6	0.9745 (5)	0.2185 (4)	0.4651 (3)	0.0352 (9)	
C7	0.9368 (5)	0.1246 (4)	0.3407 (3)	0.0374 (9)	
C8	0.6944 (4)	−0.0592 (4)	0.1789 (3)	0.0361 (9)	
C9	0.5043 (5)	−0.1312 (5)	0.1558 (3)	0.0426 (10)	
H9	0.4257	−0.1074	0.2119	0.051*	
C10	0.4338 (5)	−0.2380 (5)	0.0491 (3)	0.0415 (10)	
C11	0.5448 (5)	−0.2769 (5)	−0.0357 (3)	0.0482 (10)	
H11	0.4944	−0.3497	−0.1073	0.058*	
C12	0.7330 (5)	−0.2050 (5)	−0.0116 (3)	0.0485 (11)	
H12	0.8109	−0.2302	−0.0677	0.058*	
C13	0.8080 (5)	−0.0967 (5)	0.0939 (3)	0.0457 (10)	
H13	0.9353	−0.0485	0.1081	0.055*	
Cl1	0.19425 (13)	−0.32624 (14)	0.02129 (9)	0.0658 (4)	
N1	0.7608 (4)	0.0472 (4)	0.2912 (3)	0.0417 (8)	
H1	0.6751	0.0650	0.3345	0.050*	
N2	1.0821 (4)	0.1190 (4)	0.2841 (3)	0.0428 (8)	
O1	1.4055 (3)	0.1090 (3)	0.4010 (2)	0.0619 (8)	
O2	1.3450 (4)	0.3710 (3)	0.3184 (2)	0.0645 (8)	
S1	1.27387 (13)	0.22471 (14)	0.37119 (9)	0.0481 (3)	

### Atomic displacement parameters (Å^2^)

**Table:**

	*U*^11^	*U*^22^	*U*^33^	*U*^12^	*U*^13^	*U*^23^
C1	0.029 (2)	0.039 (2)	0.041 (2)	−0.0055 (17)	0.0023 (17)	0.0026 (19)
C2	0.044 (2)	0.052 (3)	0.047 (3)	−0.011 (2)	−0.003 (2)	−0.001 (2)
C3	0.062 (3)	0.062 (3)	0.039 (3)	−0.004 (2)	0.002 (2)	−0.002 (2)
C4	0.056 (3)	0.063 (3)	0.045 (3)	−0.001 (2)	0.013 (2)	−0.001 (2)
C5	0.036 (2)	0.059 (3)	0.041 (2)	−0.0003 (19)	0.0038 (19)	0.002 (2)
C6	0.033 (2)	0.039 (2)	0.033 (2)	0.0038 (17)	0.0032 (17)	0.0035 (19)
C7	0.028 (2)	0.037 (2)	0.043 (2)	−0.0002 (17)	0.0007 (17)	0.0000 (19)
C8	0.028 (2)	0.040 (2)	0.036 (2)	0.0022 (17)	0.0018 (17)	−0.0021 (19)
C9	0.030 (2)	0.048 (3)	0.047 (3)	−0.0001 (18)	0.0107 (18)	−0.005 (2)
C10	0.027 (2)	0.042 (3)	0.049 (3)	−0.0019 (17)	0.0015 (18)	−0.005 (2)
C11	0.040 (2)	0.054 (3)	0.044 (2)	−0.0013 (19)	0.0037 (19)	−0.010 (2)
C12	0.036 (2)	0.064 (3)	0.042 (2)	0.002 (2)	0.0083 (19)	−0.007 (2)
C13	0.024 (2)	0.065 (3)	0.044 (2)	0.0020 (18)	0.0045 (18)	0.001 (2)
Cl1	0.0325 (6)	0.0807 (9)	0.0706 (8)	−0.0122 (5)	0.0055 (5)	−0.0205 (6)
N1	0.0249 (16)	0.054 (2)	0.0411 (19)	−0.0042 (14)	0.0069 (14)	−0.0036 (16)
N2	0.0248 (16)	0.057 (2)	0.0421 (19)	−0.0024 (14)	0.0065 (14)	−0.0062 (16)
O1	0.0289 (15)	0.077 (2)	0.073 (2)	0.0088 (14)	0.0005 (13)	−0.0092 (17)
O2	0.0539 (18)	0.070 (2)	0.0632 (19)	−0.0227 (15)	0.0210 (15)	0.0020 (16)
S1	0.0261 (5)	0.0609 (8)	0.0494 (7)	−0.0073 (5)	0.0045 (5)	−0.0087 (6)

### Geometric parameters (Å, °)

**Table:**

C1—C2	1.367 (5)	C8—C9	1.389 (4)
C1—C6	1.390 (4)	C8—N1	1.417 (4)
C1—S1	1.760 (4)	C9—C10	1.374 (5)
C2—C3	1.382 (5)	C9—H9	0.9300
C2—H2	0.9300	C10—C11	1.370 (5)
C3—C4	1.371 (5)	C10—Cl1	1.746 (3)
C3—H3	0.9300	C11—C12	1.376 (4)
C4—C5	1.384 (5)	C11—H11	0.9300
C4—H4	0.9300	C12—C13	1.375 (5)
C5—C6	1.376 (4)	C12—H12	0.9300
C5—H5	0.9300	C13—H13	0.9300
C6—C7	1.477 (5)	N1—H1	0.8600
C7—N2	1.310 (4)	N2—S1	1.635 (3)
C7—N1	1.343 (4)	O1—S1	1.432 (3)
C8—C13	1.384 (4)	O2—S1	1.428 (3)
			
C2—C1—C6	122.5 (3)	C10—C9—H9	120.4
C2—C1—S1	130.4 (3)	C8—C9—H9	120.4
C6—C1—S1	107.1 (3)	C11—C10—C9	122.3 (3)
C1—C2—C3	117.7 (4)	C11—C10—Cl1	119.2 (3)
C1—C2—H2	121.2	C9—C10—Cl1	118.5 (3)
C3—C2—H2	121.2	C10—C11—C12	118.0 (3)
C4—C3—C2	120.7 (4)	C10—C11—H11	121.0
C4—C3—H3	119.6	C12—C11—H11	121.0
C2—C3—H3	119.6	C13—C12—C11	121.3 (3)
C3—C4—C5	121.3 (4)	C13—C12—H12	119.3
C3—C4—H4	119.4	C11—C12—H12	119.3
C5—C4—H4	119.4	C12—C13—C8	120.0 (3)
C6—C5—C4	118.7 (4)	C12—C13—H13	120.0
C6—C5—H5	120.7	C8—C13—H13	120.0
C4—C5—H5	120.7	C7—N1—C8	130.1 (3)
C5—C6—C1	119.2 (3)	C7—N1—H1	115.0
C5—C6—C7	131.3 (3)	C8—N1—H1	115.0
C1—C6—C7	109.5 (3)	C7—N2—S1	109.9 (2)
N2—C7—N1	122.9 (3)	O2—S1—O1	115.88 (17)
N2—C7—C6	116.9 (3)	O2—S1—N2	110.41 (16)
N1—C7—C6	120.2 (3)	O1—S1—N2	109.76 (16)
C13—C8—C9	119.3 (3)	O2—S1—C1	111.86 (17)
C13—C8—N1	123.8 (3)	O1—S1—C1	110.64 (17)
C9—C8—N1	116.9 (3)	N2—S1—C1	96.63 (15)
C10—C9—C8	119.1 (3)		
			
C6—C1—C2—C3	−0.3 (6)	Cl1—C10—C11—C12	179.7 (3)
S1—C1—C2—C3	−179.8 (3)	C10—C11—C12—C13	−0.3 (6)
C1—C2—C3—C4	0.3 (6)	C11—C12—C13—C8	0.6 (6)
C2—C3—C4—C5	−0.1 (6)	C9—C8—C13—C12	−0.5 (5)
C3—C4—C5—C6	0.0 (6)	N1—C8—C13—C12	178.3 (3)
C4—C5—C6—C1	−0.1 (5)	N2—C7—N1—C8	4.0 (6)
C4—C5—C6—C7	178.8 (4)	C6—C7—N1—C8	−174.8 (3)
C2—C1—C6—C5	0.2 (6)	C13—C8—N1—C7	−3.2 (6)
S1—C1—C6—C5	179.8 (3)	C9—C8—N1—C7	175.6 (3)
C2—C1—C6—C7	−178.9 (3)	N1—C7—N2—S1	−179.4 (3)
S1—C1—C6—C7	0.8 (4)	C6—C7—N2—S1	−0.6 (4)
C5—C6—C7—N2	−179.1 (4)	C7—N2—S1—O2	−115.4 (3)
C1—C6—C7—N2	−0.1 (4)	C7—N2—S1—O1	115.7 (3)
C5—C6—C7—N1	−0.2 (6)	C7—N2—S1—C1	0.9 (3)
C1—C6—C7—N1	178.7 (3)	C2—C1—S1—O2	−66.3 (4)
C13—C8—C9—C10	0.1 (5)	C6—C1—S1—O2	114.1 (3)
N1—C8—C9—C10	−178.8 (3)	C2—C1—S1—O1	64.5 (4)
C8—C9—C10—C11	0.2 (6)	C6—C1—S1—O1	−115.0 (3)
C8—C9—C10—Cl1	−179.6 (3)	C2—C1—S1—N2	178.6 (4)
C9—C10—C11—C12	−0.1 (6)	C6—C1—S1—N2	−1.0 (3)

### Hydrogen-bond geometry (Å, °)

**Table:**

*D*—H···*A*	*D*—H	H···*A*	*D*···*A*	*D*—H···*A*
C13—H13···N2	0.93	2.28	2.901 (5)	124
N1—H1···O1^i^	0.86	2.24	3.084 (4)	165
C5—H5···O1^i^	0.93	2.51	3.396 (4)	159
C2—H2···O2^ii^	0.93	2.42	3.302 (4)	158

Symmetry codes: (i) *x*−1, *y*, *z*; (ii) −*x*+3, −*y*+1, −*z*+1.

## References

[bb1] Bernstein, J., Davis, R. E., Shimoni, L. & Chang, N.-L. (1995). *Angew. Chem. Int. Ed. Engl.* **34**, 1555–1573.

[bb2] Brigas, A. F., Clegg, W., Dillon, C. J., Fonseca, C. F. C. & Johnstone, R. A. W. (2001). *J. Chem. Soc. Perkin Trans. 2*, pp. 1315–1324.

[bb3] Bruker (2005). *SADABS* Bruker AXS Inc., Madison, Wisconsin, USA.

[bb4] Bruker (2007). *APEX2* and *SAINT* Bruker AXS Inc., Madison, Wisconsin, USA.

[bb5] Farrugia, L. J. (1997). *J. Appl. Cryst.* **30**, 565.

[bb6] Farrugia, L. J. (1999). *J. Appl. Cryst.* **32**, 837–838.

[bb7] Rafique, M., Hussain, G., Siddiqui, W. A. & Tahir, M. N. (2009). *Acta Cryst.* E**65**, o1883. 10.1107/S1600536809027007 PMC2977349 21583576

[bb8] Sheldrick, G. M. (2008). *Acta Cryst.* A**64**, 112–122. 10.1107/S0108767307043930 18156677

[bb9] Siddiqui, W. A., Ahmad, S., Siddiqui, H. L., Hussain, T. & Parvez, M. (2010). *J. Chem. Crystallogr.* **40**, 116–121.

[bb10] Spek, A. L. (2009). *Acta Cryst.* D**65**, 148–155. 10.1107/S090744490804362X PMC2631630 19171970

